# Anodal direct current stimulation of the auditory cortex at the onset of presbycusis delays cortical aging

**DOI:** 10.1007/s00429-025-02912-w

**Published:** 2025-04-25

**Authors:** I. S. Fernández del Campo, A. J. de la Fuente, I. Díaz, I. Plaza, M. A. Merchán

**Affiliations:** https://ror.org/02f40zc51 grid.11762.33 0000 0001 2180 1817 Lab.4 Auditory Neuroplasticity, Institute for Neuroscience of Castilla y León, University of Salamanca, Salamanca, Spain

**Keywords:** Wistar rat, Natural aging, Cortical excitability, Excitatory/inhibitory balance, Age related hearing loss

## Abstract

Presbycusis or age-related hearing loss (ARHL) affects millions of people worldwide, increasing their risk of cognitive decline and poor quality of life. However, ARHL remains an irreversible condition due to our inability to induce inner-ear hair cell regeneration. Nevertheless, multisession epidural stimulation of the auditory cortex (AC) at the onset of ARHL prevents hearing threshold elevation in naturally aging Wistar rats. Accordingly, we hypothesized that anodal direct current (DC) stimulation of the AC may also compensate for age-related maladaptive, activity-dependent changes. Here, we examined immunocytochemical markers in the AC, including early genes (c-fos and Arc), AMPA receptors (GluR2/3), parvalbumin (PV), and GAD67, along with auditory-evoked potentials (CAEPs) recorded in both auditory and visual (VC) cortices. When comparing 6 and 18.13-month-old rats without AC simulation, we observed loss of c-fos and Arc-positive neurons and decreased GluR2/3 expression, confirming altered AC neuronal network plasticity and activation. In addition, we noted changes in PV and decreased GAD67 immunoreactivity suggesting disrupted inhibition and significantly increased wave amplitudes in CAEPs, altered AC latencies, and decreased VC responses. By contrast, electrically stimulated rats showed no significant variations in early gene markers, GluR2/3, PV, or GAD67 with age, and the amplitudes and latencies of CAEPs recorded in their AC and VC resembled those of young rat. These findings indicate that anodal DC stimulation at the onset of ARHL delays AC aging by minimizing the loss of inhibition and preventing increases in cortical excitability in Wistar rats.

**Supplementary Information:**

The online version contains supplementary material available at 10.1007/s00429-025-02912-w.

## Introduction

Physiological aging is characterized by mitochondrial dysfunction and intracellular accumulation of reactive oxygen species (ROS), decreased energy metabolism, dysregulation of neuronal network activity, and inflammation in the brain cortex (Mattson, Mark P. and Arumugam 2018). This decrease in energy supply to neurons with aging adversely affects energy-dependent functions, such as neurotransmission and ionic gradients, impairing synaptic plasticity (Zia et al. 2021). Intracellular accumulation of ROS has been also linked to microglia activation and increased expression of inflammatory mediators (Pawate et al. 2004; Di Stadio and Angelini 2019). Activity-dependent changes following presbycusis have been proposed as the origin of the loss of inhibition and increased excitability in the AC during aging (Schmidt et al. 2010; Herrmann and Butler 2021).

Age related hearing loss (ARHL) is one of the most prevalent chronic sensory deficit experienced by older adults, contributing to social isolation, cognitive impairment and neurodegenerative brain disorders (Gates and Mills 2005; Gates et al. 2010; Juiz et al. 2023). But given the lack strategies for hair cell regeneration, therapeutical approaches for ARHL have long been limited to preserving hearing by protecting inner ear and auditory nerve cells against damaging factors and by preventing risk factors, such as sound overexposure and ototoxicity (Kujawa and Liberman 2019; Joo et al. 2020). Nevertheless, AC electric stimulation can delay the onset of ARHL in naturally aging Wistar rats by promoting efferent system plasticity through activation of the descending cortico-olivary pathway, as shown in our recent studies (Fernández del Campo et al. 2024; Fuentes-Santamaría et al. 2024). Therefore, understanding the underlying mechanism of electric stimulation-induced ARHL delays requires a deeper analysis of the brain cortex.

In the rat cortex, polarizing currents induce long-lasting effects on electrical activity (Bindman et al. 1964). Furthermore, direct current (DC) anodal stimulation increases neuronal responses in both pyramidal and non-pyramidal neurons (Purpura and McMurtry 1965). In humans, such activating effects of anodal DC stimulation have been observed in recordings of cortical auditory evoked potentials (CAEPs) (Zaehle et al. 2011). Corroborating these results, our multisession DC stimulation protocol induces a sustained, reversible threshold elevation measured with ABRs (Colmenárez-Raga et al. 2019). These findings provide indirect evidence of long-lasting cortical activation.

In the AC of 22 month-old Fischer rats, both mRNA and protein expression of enzymes directly involved in GABA synthesis (GAD65&67) decrease significantly, as shown by in situ hybridization and immunocytochemistry, respectively (Ling et al. 2005). In the AC of aged Long-Evans rats, GAD65&67 protein levels are also downregulated, as shown by Western Blot (Burianova et al. 2009). In line with these results, the AC of aged Fischer 344 rats display loss of parvalbumin-immunoreactive neurons, widely accepted as GABA interneurons (Ouda et al. 2008). In this rapidly aging rat strain, CAEPs display increased wave amplitudes upon ARHL, suggesting that loss of inhibition may underline increased cortical responses to sound (Schmidt et al. 2010; Herrmann and Butler 2021). By compensating by anodal stimulation for the altered lemniscal activation of the AC, induced by cochlear input loss, anodal stimulation may thus delay the onset of ARHL.

Considering the above, we hypothesize that sustained anodal DC stimulation of the AC may help to maintain neuronal network activation and offset maladaptive activity-dependent changes caused by aging. To test this hypothesis, we compared early gene (c-fos and Arc), AMPA receptor (GluR2/3), parvalbumin (PV) and GAD67 immunocytochemistry markers and CAEP recorded in the auditory and visual (VC) cortices of young (6 months) and old (17.13 months) Wistar rats subjected or not to electrical stimulation of the AC. Leveraging sustained anodal DC stimulation to counteract age-related auditory cortical dysfunction may pave the way for targeted neuromodulatory therapies aimed at delaying the onset and/ or slowing down ARHL the progression.

## Materials and methods

This study was conducted in compliance with Spanish regulations (Royal Decree 53/2013—Law 32/2007) and European Union guidelines (Directive 2010/63/EU) on animal care and use in biomedical research. All surgeries were performed under monitored anesthesia (respiratory rate, body temperature, and oxygen saturation), making all efforts to minimize suffering.

### Experimental groups

**Fig. 1:**
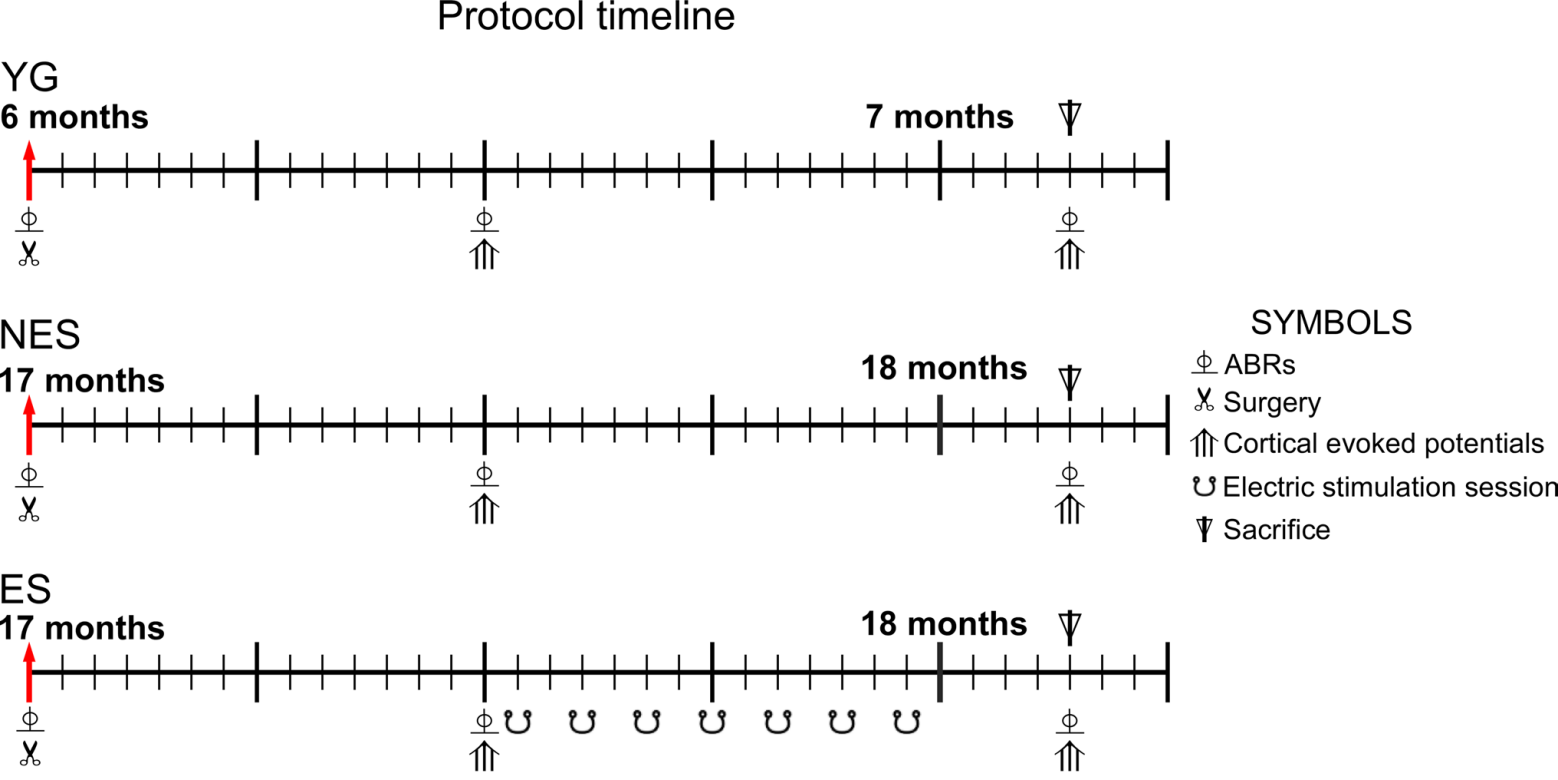
Experimental paradigm (protocol timeline). Red arrows label the age at the start of the protocol, (time point of the life span at the first intervention-surgery). In the ES group, electric stimulation was initiated at 17.5 months (525 days), and the rats were euthanized at 18.13 months (544 days). Animal groups: control (YG), non-stimulated (NES) and stimulated (ES) rats. Symbols indicate the time point of the recordings, electric stimulation and euthanasia during the protocol

Eighteen, male rats were used in three experimental groups (Fig. 1):

- Young, six-month controls (YG): n = 6.
- Aged, 17-month-old non-stimulated (NES): n = 6.
- Aged, 17-month-old and electrically stimulated (ES): n = 6.

In our experimental design, we used only male rats because they are more resistant to surgery. However, male and female rats differ in the onset of presbycusis and in brain aging due to hormonal effects. Therefore, these results must be confirmed in female rates for a conclusive validation of our main findings, regardless of sex differences (Balagova et al. 2018).

### ABRs

Recordings were conducted under gas anesthesia using a real-time signal processing system RZ6 Multi I/O Processor (Tucker-Davis Technologies (TDT), Alachua, Fl, United States). Before recording, sound system outputs were calibrated using a one-quarter-inch microphone (Brüel and Kjaer). The sound stimuli consisted of 0.1-ms alternating polarity clicks, delivered at a repetition rate of 21 clicks/s and presented in 10 dB SPL ascending steps, ranging from 10 to 90 dB SPL. The stimulation sessions were performed in an acoustically isolated chamber, delivering stimuli to the ear through a 10-cm-long plastic tube via an MF1 multi-field magnetic speaker (TDT) in close field. With this method, the total delay in stimulus arrival to the tympanic membrane was 1.4 ms.

ABRs were recorded using three subcutaneous needle electrodes placed at the vertex and both mastoids, averaging 1,000 electroencephalogram (EEG) responses to 1000 stimuli. The AEPs were then amplified and digitized using a Medusa TA16PA preamplifier and a RA4LI head stage (TDT). Monaural ABRs were recorded from the vertex, using the electrode on the mastoid ipsilateral to the stimulated ear as reference and the electrode in the contralateral mastoid to the stimulated ear as the ground. Monaural ABRs were sequentially recorded in both ears and analyzed separately. The final signal was filtered using a 500-Hz high-pass filter and a 3.000-Hz low-pass filter.

Waves were initially recorded in ABRs, and thresholds were calculated using the software Matlab (©MatLab R- 2017 a). ABR thresholds were defined as the minimum sound intensity that evoked a significant voltage change in latency ranging from 1.4 to 5 ms and exceeding the mean ± 2 standard deviations of the voltage of background activity after the first 1.4 ms of the recording.

### Surgery

Animals were anesthetized with 2.5% isoflurane and placed in a stereotaxic frame to expose the left temporal cranial surface. The Paxinos and Watson stereotaxic coordinates (Paxinos and Watson 2005) were used to locate the AC coordinates (IA: 4.08 mm) on the bone surface. Then, 1-mm holes were drilled at these coordinates, with cold saline (4 °C) applied to prevent thermal cortical lesions. A 1-mm silver ball electrode was embedded in the trepan. Two screws, 1 mm in diameter, were inserted on the contralateral side (IA: 3.35 mm) and in the most rostral area of the skull (IA: 11.04 mm) (ground). The electrode and screws were glued to the skull surface and completely covered with dental cement. Once the silver ball electrode was placed, a consistent surgical approach was followed across all three animal groups, including the YG (sham-operated) control group. After euthanasia, skulls were extracted and fixed to assess the depth of the embedded electrodes and to ensure that the meninges remained intact. Potential tissue damage caused by the electrodes was ruled out in a previous study (see Colmenárez-Raga et al. 2019) with a similar protocol to that applied in the present study.

### Cortical auditory evoked potentials (CAEPs)

Recordings were performed in a sound-attenuating, electrically shielded room, with the animals under gas anesthesia (isoflurane) and placed in a stereotaxic frame. Body temperature was maintained at 37 °C using a homeothermic blanket system with a rectal thermometer probe. Then, 16-kHz, 50-ms, 50-dB pure tone auditory stimuli were generated using a TDT system 3, and the cortical signal was amplified (50x), 3 Hz-high-pass and 3 kHz-low-pass filtered using a differential amplifier (Model 1700 A-M System, LLC, Carlsborg, WA, USA), and continuously sampled at 5 kHz by a 1401 CED A/D converter card (Cambridge Electronic Design, UK) managed in Spike2 software (Cambridge Electronic Design, UK, version 2.8). The digitized data were stored in a PC for subsequent analysis. Each session consisted of 100 iterations of a basic 3-s cycle with one auditory stimulus (see above). The recordings were made first on the electrode placed over the AC and then on the electrode located over the VC. The reference electrode was placed rostrally, around the vertex.

### DC stimulation protocol

An ISU 200 BIP isolation unit controlled by a CS20 stimulator (Cibertec, Madrid, Spain) was used to deliver a 0.1-mA continuous current for 10 min per session though the epidural bone-attached electrode (anode). The voltage current was monitored for stability during all stimulation sessions. Electrical stimulation was applied in awake animals for seven sessions on alternate days (for more details, see: Colmenárez-Raga et al. 2019).

### Fixation and sectioning

At 8.13 months in the YG group and at 18.13 months in the NES and ES groups (Fig. 1), animals were deeply anesthetized by intraperitoneal injection of 6% sodium pentobarbital (60 mg/Kg BW) and transcardially perfused with 4% p-formaldehyde in 0.1 M phosphate buffer (PB). All brains were dissected and post-fixed for 48 h in the same solution of 4% p-formaldehyde before cryoprotection by immersion in 30% sucrose in 0.1 M PB, pH 7.4 at 4 °C for 48 H. The brains were carved and serially sectioned (40 µm thickness) in the coronal plane with a sliding freezing microtome (HM 430 Sliding, MICROM International, Waldorf, Germany).

### Immunostaining

Alternate coronal serial sections were immunostained for c-fos, Arc/Arg3.1., GluR2/3, Parvalbumin and GAD67 (Table 1). Free-floating sections were washed with 0.1 M PB pH 7.6, followed by endogenous peroxidase inhibition induced by incubation with 10% methanol + 3% H_2_O_2_ in 0.1 M PB for 20 min. Subsequently, the sections were washed in 0.1 M PBS and 0.05 M TBS-Tx, pH 8.0, 0.3% Triton X-100 (T9284 Sigma, St. Louis, MO, USA; TBS-Tx) and incubated with the primary antisera (Table 1). Nonspecific labeling was blocked in 10% fetal calf serum. After washing three times with TBS-Tx for 15 min, the sections were incubated with an anti-rabbit biotinylated secondary antibody (biotinylated anti-rabbit IgG H + L, BA-1000; Vector, Burlingame, CA, USA) at a 1:200 dilution in TBS-Tx for 120 min, at room temperature. The sections were then washed with TBS-Tx and incubated for 180 min in avidin/biotin peroxidase (ABC complex, Vectastain Standard ABC kit PK-4000; Vector, Burlingame, CA, United States) and further washed with TBS-Tx, followed by Tris–HCl, pH 8.0. These sections were then incubated in 3.3 diaminobenzidine tetrahydrochloride (DAB; d-9015; Sigma-Aldrich, St. Louis, MO, United States) with 0.006% H_2_O_2_ and 0.4% nickel ammonium sulfate to visualize the peroxidase reaction. Processed without the primary antibody, negative controls were performed to confirm immunostaining specificity.

**Table 1:** Summarized description of antibodies used

Antigen	Immunogen	Description	Dilution used
c-fos	Synthetic peptide corresponding to AA 2 to 17 from rat c-fos (UnitProt Id: P12841)	Polyclonal rabbit, Synaptic Systems Cat # 226,003, RRID:AB_2231974	1:1000 TBS 0.05 M 1 Triton-Tx 0.3%
Arc/Arg3.1	Strep-Tag® fusion protein of full-length mouse ARC. Enriched synaptosome fraction of rat brain(P2)	Polyclonal Rabbit, Synaptic Systems 156,003; RRID:AB_887694	1:1,000 TBS 0.05 M + Triton-Tx 0.3%
GluR2/3	Carboxy terminus peptide of rat GluR2 conjugated to BSA with glutaraldehyde (EGYNVYGIESVKI)	Polyclonal Rabbit, Merck Millipore #AB1506; RRID:AB_90710	1:100 TBS 0.05 M + Triton-Tx 0.3%
Parvalbumin	Frog muscle parvalbumin	Monoclonal mouse Sigma-Aldrich Cat# P3088, RRID:AB_477329	1:1000 TBS 0.05 M + Triton-Tx 0.3%
GAD67	Recombinant GAD67 protein	Monoclonal mouse, Merck Millipore #MAB5406 clone 1G10.2 RRID:AB_2278725	1:1000 TBS 0.05 M + Triton-Tx 0.3%
Iba 1	C-terminus of Iba l (‘NPTGPPAKKAISELPC’)	Polyclonal rabbit, Wako Cat # 019–19741, RRID:AB_839504	1:1000 TBS 0.05 M 1 Triton-Tx 0.3%

### Morphometric and densitometric analysis

Immunostained sections were analyzed by acquiring high-resolution microphotographs (digital resolution of 132 pixel/100 μm^2^) at 10X magnification under a Leica DMRX microscope with an MBF camera (MBF Bioscience CX9000; Williston, VT, USA) to build whole-section digital mosaics in Neurolucida software (NL-Vs 8.0, MicroBrightField®, Inc., Williston, VT, USA). Photographs of fixed-reference density values were taken using a stepped density filter (11 levels; ®EO Edmund industrial optics-ref 32,599, Karlsruhe, Germany) to set homogeneous microscopic illumination conditions and to calibrate optical density (OD) measurements. For c-fos, Arc and PV immunocytochemistry, mosaics were analyzed in Image J software by maximum entropy thresholding segmentation. The mean and standard deviation of gray levels were assessed throughout the areas of analysis to cancel out differences in immunostaining intensities in and between experiments (normalized OD values). The number of segmented particles was normalized to 10.000 µm^2^. To overcome difficulties in the segmentation of immunostaining patterns involving a dense neuropil or highly populated terminal axon fields (such as PV), we performed OD analysis by selecting a frame of the Au1 from four sections per case at defined stereotaxic coordinates.

### Statistical analysis

Statistical analysis was performed using the IBM SPSS software, version 26 (IBM Corp. and SPSS Inc., Chicago, IL, United States, RRID: SCR_002865). Differences in OD, area and number of immunoreactive neurons were analyzed for c-fos, Arc and PV quantitative immunocytochemistry. Where appropriate, Kolmogórov-Smirnov and Shapiro-Wilks tests were used to analyze the normal distribution of the data. Subsequently, an unpaired *t* test was performed for each marker to assess differences between and right cortices, and one-way analysis of variance (ANOVA) followed by Bonferroni and HSD Tukey post-hoc tests were performed to identify statistical differences in OD, area and number/10.000 μm^2^ of immunoreactive neurons between groups. For CAEPs, differences in peak-to-peak and latency values of P1-N1 and P2-N2 between groups were determined using the non-parametric Friedman test followed by Bonferroni post hoc, while the Mann–Whitney test was used for comparisons between groups at a single recording time. The significance level was set at *p* < 0.05. The results are expressed as mean ± SEM.

## Results

### c-fos Immunocytochemistry

**Fig. 2:**
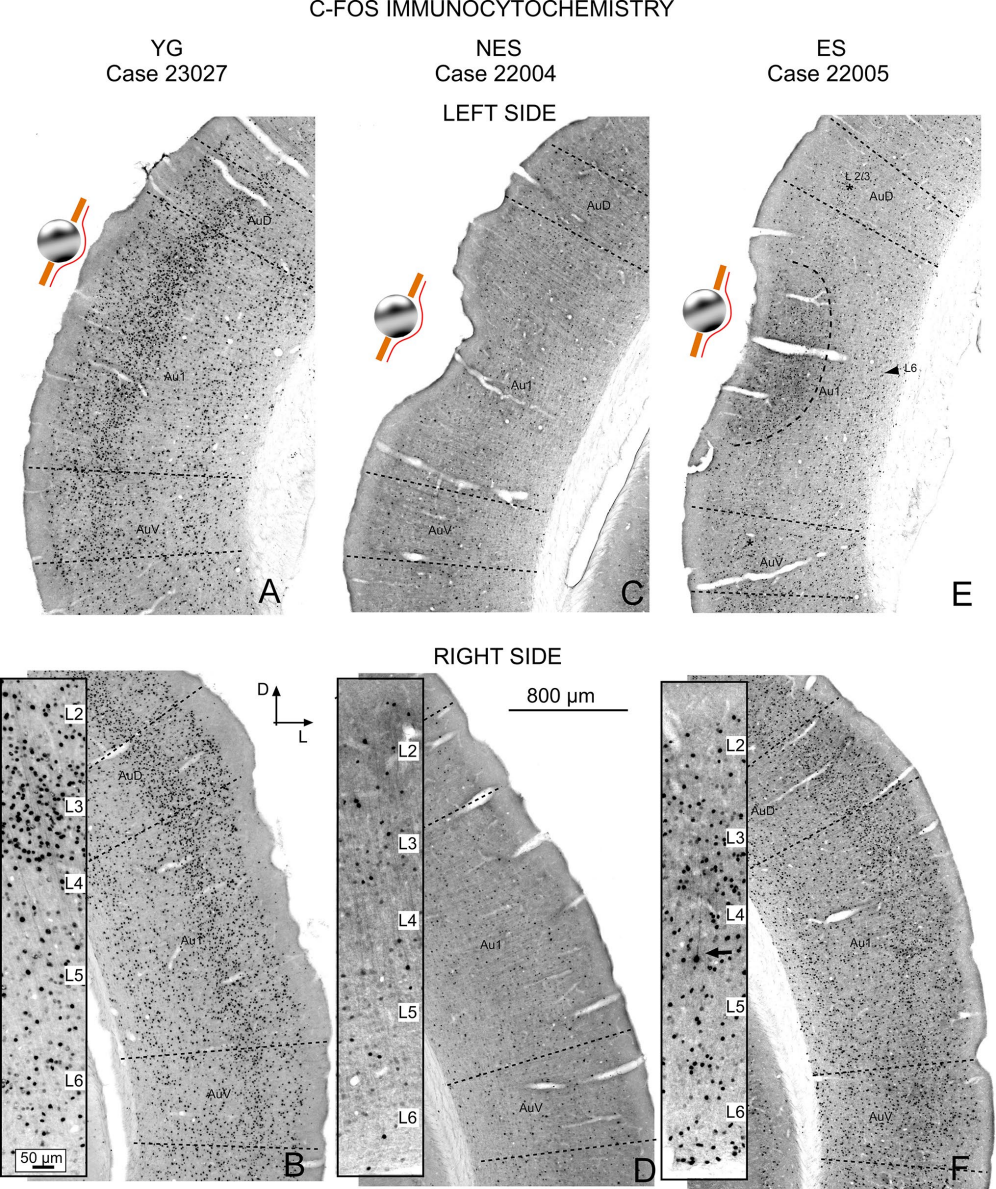
c-fos immunocytochemistry. **A** YG. Area of sham electrode implantation in the left AC (the sphere indicates the tentative location of the electrode). **B** YG. Right contralateral AC at the same coordinates. On both sides, the AC shows well-defined layers with a higher concentration of positive cells in layer 3 (inset). **C** NES left side. Electrode implantation area. **D** NES right side. In this experimental group, a few positive neurons are dispersed along the AC bilaterally. Although the layering is not well defined in panoramic views, the higher number of cells at a higher magnification outlines layer 3 (inset). **E** ES left side. Electric activation area. The surface area of the AC shows loss of layering and an increased number of particles (dotted lines). Layers can be easily distinguished outside the stimulation area. **F** ES right side shows dense immunoreactive cells. Inset. Arrow pointed an overstained pyramidal cell of layer 5. In **B**, **D** and **F**, the insets have been taken at a higher magnification of the central part of A1 from the same section of the panoramic view

In the control group (YG), the highest concentration of c-fos-positive cells of the AC was found in the supra-granular layer 3 (Fig. 2A and B inset). Scattered, less abundant positive cells were observed in layers 5 and 6 (Fig. 2A and B inset). This canonical c-fos cytoarchitectural distribution was well preserved around the electrode implantation area in the left AC (compare Fig. 2A and B). Although we were unable to visualize dendritic neuronal trees in our material, the size, density, and distribution of c-fos-positive neurons revealed that different neuronal types were labelled throughout the AC (Fig. 2B, inset).

Bilaterally, NES rats showed fewer c-fos positive neurons than YG rats across all layers of the AC (implanted – left AC; not implanted – right AC) (Fig. 2C and D). The global decrease in immunolabeled cells prevented a clear definition of the cytoarchitectural layering of the cortex (Fig. 2D inset).

In ES rats, the electrode implantation site was topographically identified in sections by a small depression in the surface of the cortex (Fig. 2C and E). In these animals, the electrode implantation site was associated with a high concentration of c-fos-positive cells and with loss of the cortical layering cytoarchitecture in a superficial band, roughly associated with layers 1 to 4 (Fig. 2E dotted line). Outside this superficial band, cell counts and cortical layering remained unchanged in the deep A1 cortex (Fig. 2E arrowhead L6), in the surrounding areas of the secondary ACs (Fig. 2E asterisk), and in the right non-implanted cortices (Fig. 2F). As in YG, layer 3 showed more abundant labelling than infragranular layers 5 and 6, with some overstained neurons, particularly in the right, non-implanted side (Fig. 2F inset -arrow).

**Fig. 3:**
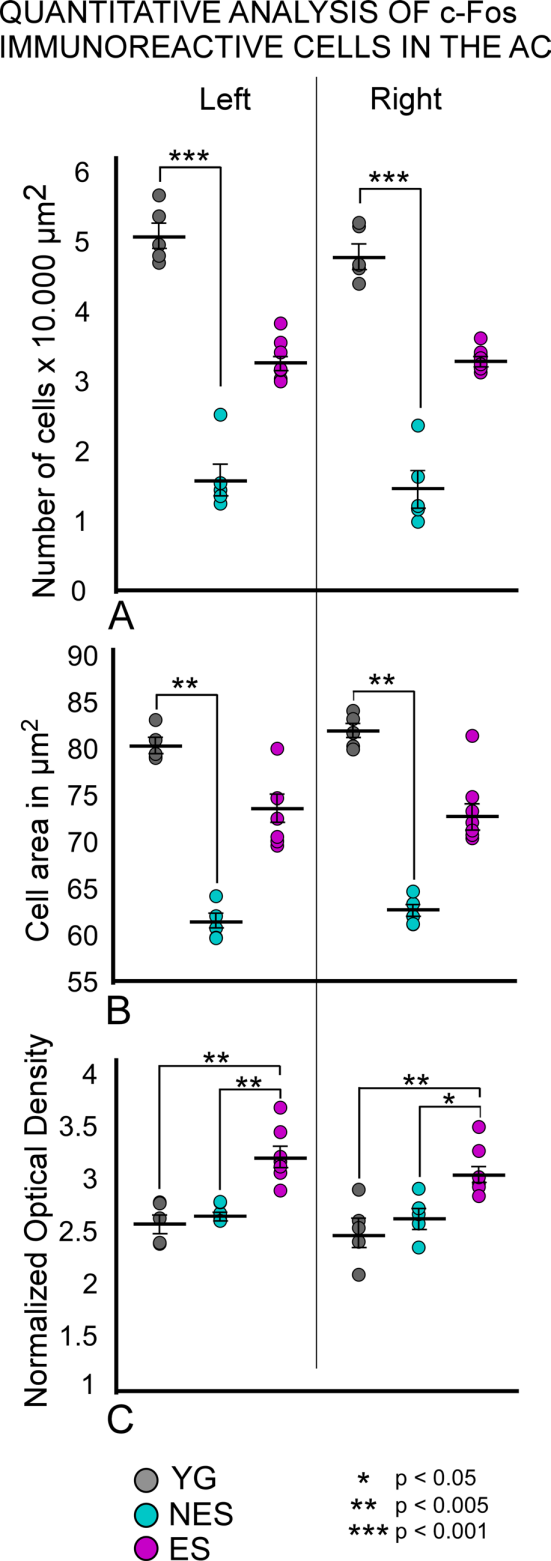
Statistical analysis of quantitative c-fos immunocytochemistry was performed by comparing the area and the normalized cells counts, and optical density of the AC between the three experimental groups. Color code: YG gray; NES cyan and ES magenta. **A** Bilaterally, the number of immunoreactive cells is significantly lower in the NES group than in the YG group. No significant differences are detected when comparing ES with the other two experimental groups. **B** The mean of positive cells is significantly lower in NES than in YG, but not in ES. **C** Bilaterally, the normalized OD values are significantly lower in YG and NES than in ES

The early gene c-fos has been widely used to detect changes in neuronal activation after stimulation as its expression is linked to CREB phosphorylation and transcription (Sheng and Greenberg 1990). Our ability to detect changes in c-fos immunoreactivity changes in the nervous system has improved through quantification and statistical analysis of parameters such as cell count, nuclear size, and reaction product density (Clarkson et al. 2010). In addition, brain mapping using quantitative c-fos immunocytochemistry now enables us to assess the topography of electric stimulation effects (Carmona-Barrón et al. 2023). In this study, we found a significantly higher number of c-fos immunoreactive neurons in YG than in NES animals, in both the ipsilateral (p = 0.001) and contralateral (p = 0.001) sides, at the electrode implantation site (Fig. 3A). Conversely, no significant differences were found between YG and ES animals on either side (p = 0.127 in the ipsilateral side and p = 0.125 in the contralateral side) (Fig. 3A). Bilaterally, c-fos-immunoreactive cell bodies from NES rats had a significantly smaller area than YG and ES cells (Fig. 3B), but these differences were only significant when comparing YG and NES groups (p > 0.023 in the ipsilateral side and p > 0.031 in the contralateral side) (Fig. 3B). When comparing normalized OD, ipsilateral cells side of ES animals were significantly darker than those of YG (p > 0.002) and NES (p > 0.003) animals, most likely as a direct result of activation by electric stimulation. In the contralateral side, ES rats also showed darker cells than YG and NES rats (Fig. 3C), with significant differences between YG (p > 0.004) and NES (p > 0.023) animals (Fig. 3C). Statistical analysis did not show significant differences in the number of cells, area and OD between ipsilateral (right) and contralateral (left) cortices in any of the experimental groups (Fig. 3).

### Arc immunocytochemistry

**Fig. 4:**
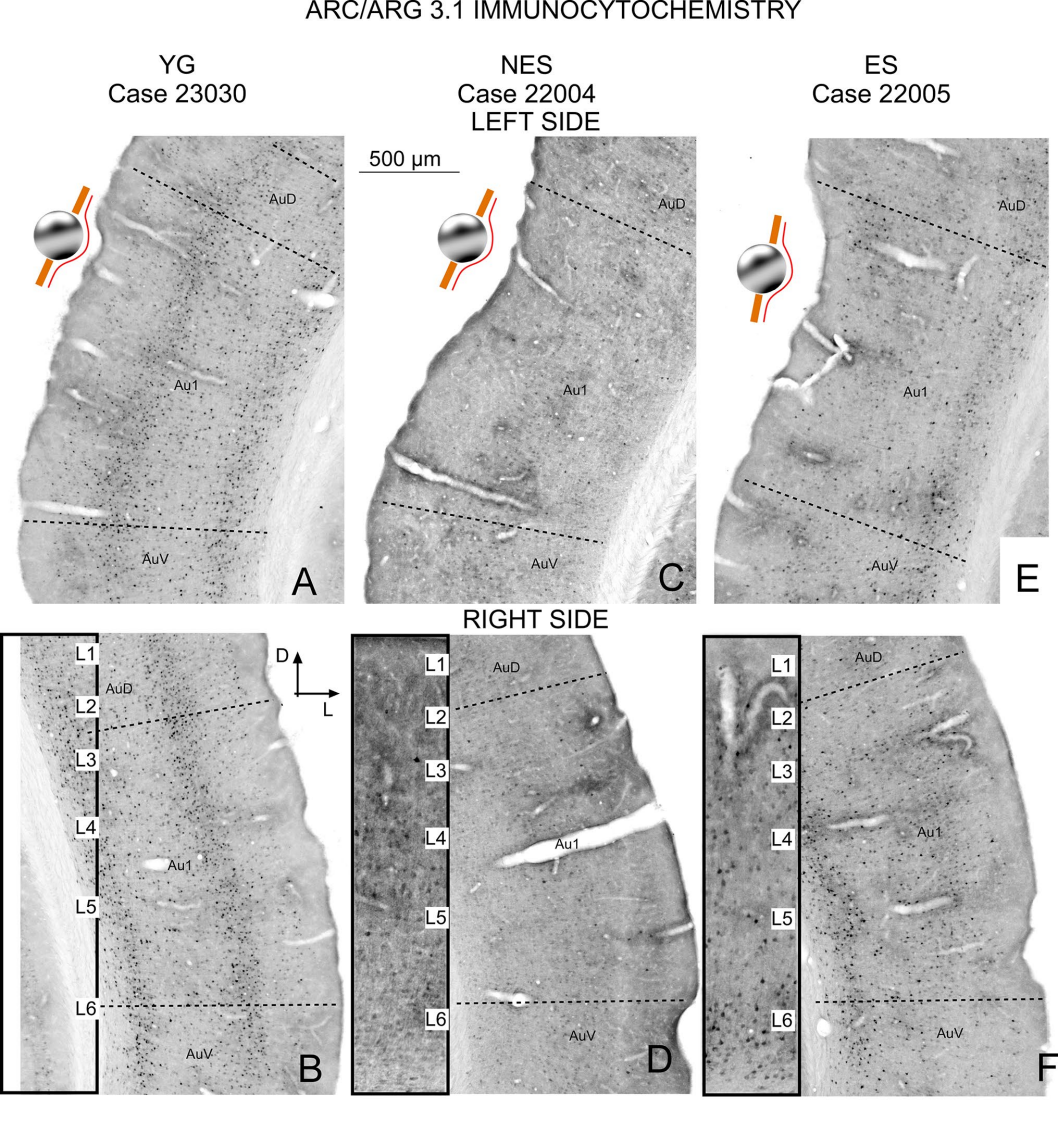
Arc/Arg3.1 immunocytochemistry. **A** YG left side. Layers 2,4 and 6 are well defined. **B** YG right side. Positive neurons outline cortical layers. The inset at a higher magnification shows that the reaction product labels cell bodies and dendrites, especially in layer 6. **C** NES left side. Few labeled neurons can be detected in the electrode implantation area. **D** NES right side. Only a few dispersed immunoreactive neurons can be seen in this group. The inset shows diffuse labeling in layers 4 and 6. **E** ES left side. Surface areas of the cortex overlapping with the electrically stimulated zone show few immunoreactive neurons, with positive neurons in infragranular layers 4, 5 and 6. **F** ES right side. Neurons distributed along all layers are concentrated in layer 6. Inset: neurons in layer 6 show strong immunoreactivity

In the left (electrode implantation site) AC of YG animals, Arc-labelled cells were detected throughout the cortex, with a higher concentration in layers 4 and 6 (Fig. 4A and B). In the right AC, a slightly higher number of immunoreactive cells was observed, albeit with the same cytoarchitectural distribution (Fig. 4B). NES rats showed markedly fewer immunoreactive neurons than YG rats, reflecting a strong global decrease in the number of Arc immunoreactive cells in all layers of the AC, on both sides (Fig. 4C and D).

ES animals showed more immunoreactive cells in the AC than NES animals, but not as many as YG animals (compare Fig. 4). Unlike NES, ES rats displayed a well-defined cortical layering organization even though the distribution of cells was slightly different from that of YG animals, with more cells concentrated in layers 2/3 and 6, and with fewer cells in layer 4 (compare insets between groups in Fig. 4). Differences between left and right ACs were also observed in these animals. Across all cortical layers, fewer cells were visible on the left side, where the electric stimulation was applied, that is, the left side (Fig. 4E and F).

**Fig. 5:**
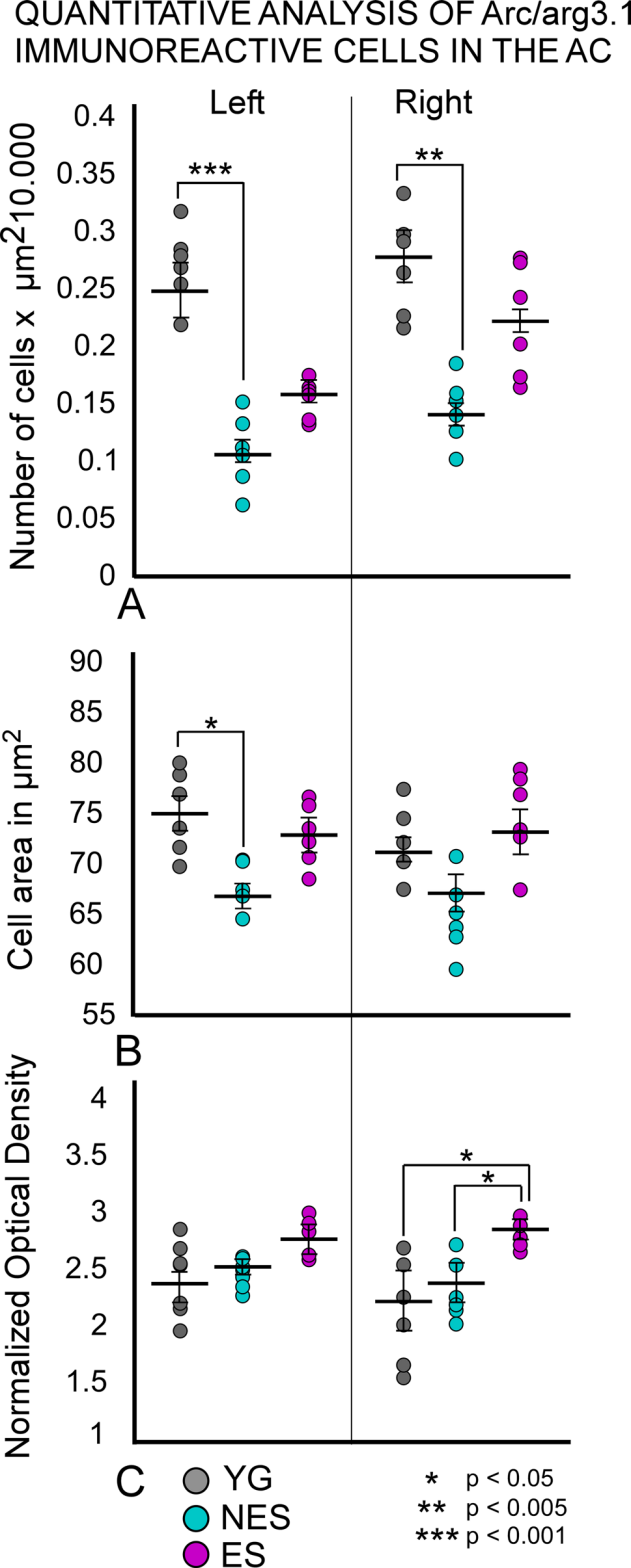
Quantitative analysis of Arc immunoreactivity: **A** Number of cells, **B** Cell area, and **C** Normalized OD. YG animals show a significant lower cell number and area than NES animals. The ES group shows nonsignificantly higher cell number and area values than the NES group. No significant differences are found when comparing left and right OD values either (data not shown), but the OD values on the right side are significantly higher in the ES group than in the YG and NES groups

Confirming our microscopic observations, morphometric analysis of immunoreactive sections showed that the number of immunoreactive cells was significantly lower in the NES group, in both the right (p = 0.001) and left (p = 0.003) sides. In addition, cell area was also significantly (p = 0.023) decreased in the left side (Fig. 5). In turn, cell density decreased more on the right than on the left side of NES rats. However, no significant differences were found when directly comparing left and right density values in any group (data not shown, YG p = 0.43, NES p = 0.37, ES p = 0.07) (Fig. 5C). The values of cell number and area were higher in the ES group than in the NES group, albeit nonsignificantly (Fig. 5A, B). Conversely, in the contralateral right side, OD values were significantly higher in the ES group than in the YG (p = 0.028) and NES (p = 0.021) groups, further confirming our microscopic observations (see above) (Fig. 5C). Statistical analysis did not show significant differences in the number of cells, area and OD between ipsilateral and contralateral cortices in any of the experimental groups (Fig. 5).

### Glu 2/3 immunocytochemistry

**Fig. 6:**
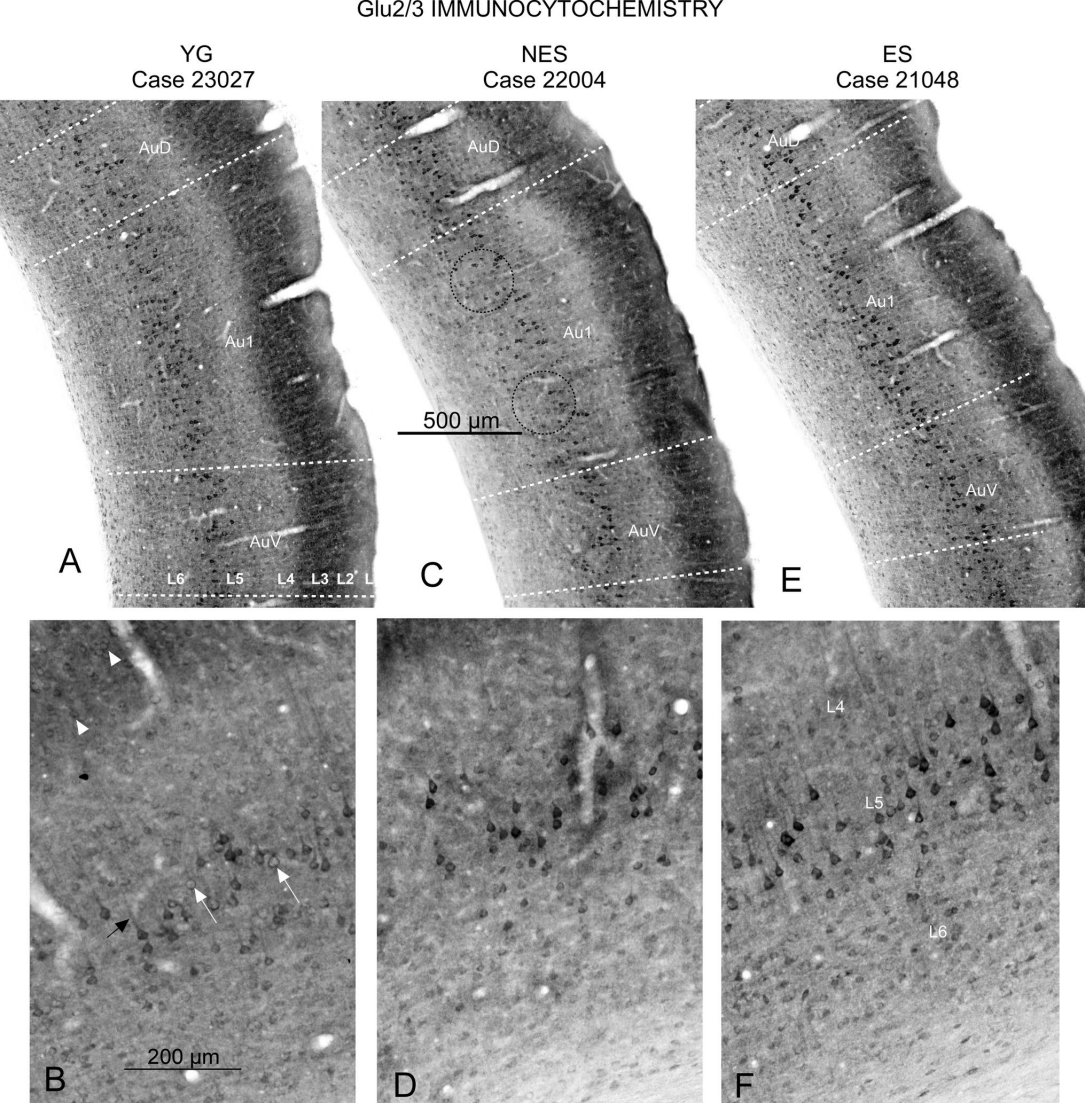
Glu2/3 AMPA receptors subunit immunocytochemistry. **A** YG. AC show pyramidal neurons with dense labeling in layers 2/3, 5 and 6. **B** YG. At a higher magnification, the hypolemmal distribution of the reaction product is identified in the cell bodies of small and medium neurons of layer 2/3 (arrowheads). The reaction product delineates cell bodies (white arrow) and dendrites (black arrow) in large pyramidal neurons of layer 5. Layer 6 shows a high concentration of positive cells with less dense but nevertheless well-defined immunostaining. **C** NES. Immunoreactive cells along layers 2/3, 5 and 6 show areas devoid of neurons in layer 5 (dotted circles) and a low-density immunoreactivity in layer 6. **D** NES. At a higher magnification, loss of immunoreactivity is observed in dendrites, as well as a blurred hypolemmal distribution of the reaction product. **E** ES. A global increase in immunoreactivity density and cell counts is detected in all positive layers. **F** ES. Detail of layer 5 neurons showing stronger immunoreactivity across all layers, including layer 4. Note the extremely dense reaction product in the cell bodies and dendrites of layer 5 neurons. Layer 6 neurons also show strong immunoreactivity

In the AC of YG rats, Glu 2/3 immunoreactivity was high in small and medium pyramidal neurons of layer 2/3 and in cell bodies of large pyramidal neurons if layers 5 and 6 (Fig. 6A and B). In YG neurons of layers 5 and 6, the reaction product clearly outlined the membrane in cell bodies and dendrites (Fig. 6B). In NES animals, fewer large and medium pyramidal neurons were stained in layers 5 and 6 (Fig. 6C dotted circles and D). Immunoreactive cells were lighter, as shown by the loss of staining in cell bodies and in dendrites (Fig. 6D). ES animals showed the densest neuronal immunostaining and more abundant immunoreactive cells, particularly along layers 5 and 6 (Fig. 6E). Pyramidal neurons displayed packed, dense reaction products in cells bodies and highlighted apical dendrites (Fig. 6F).

### Parvalbumin immunocytochemistry

**Fig. 7:**
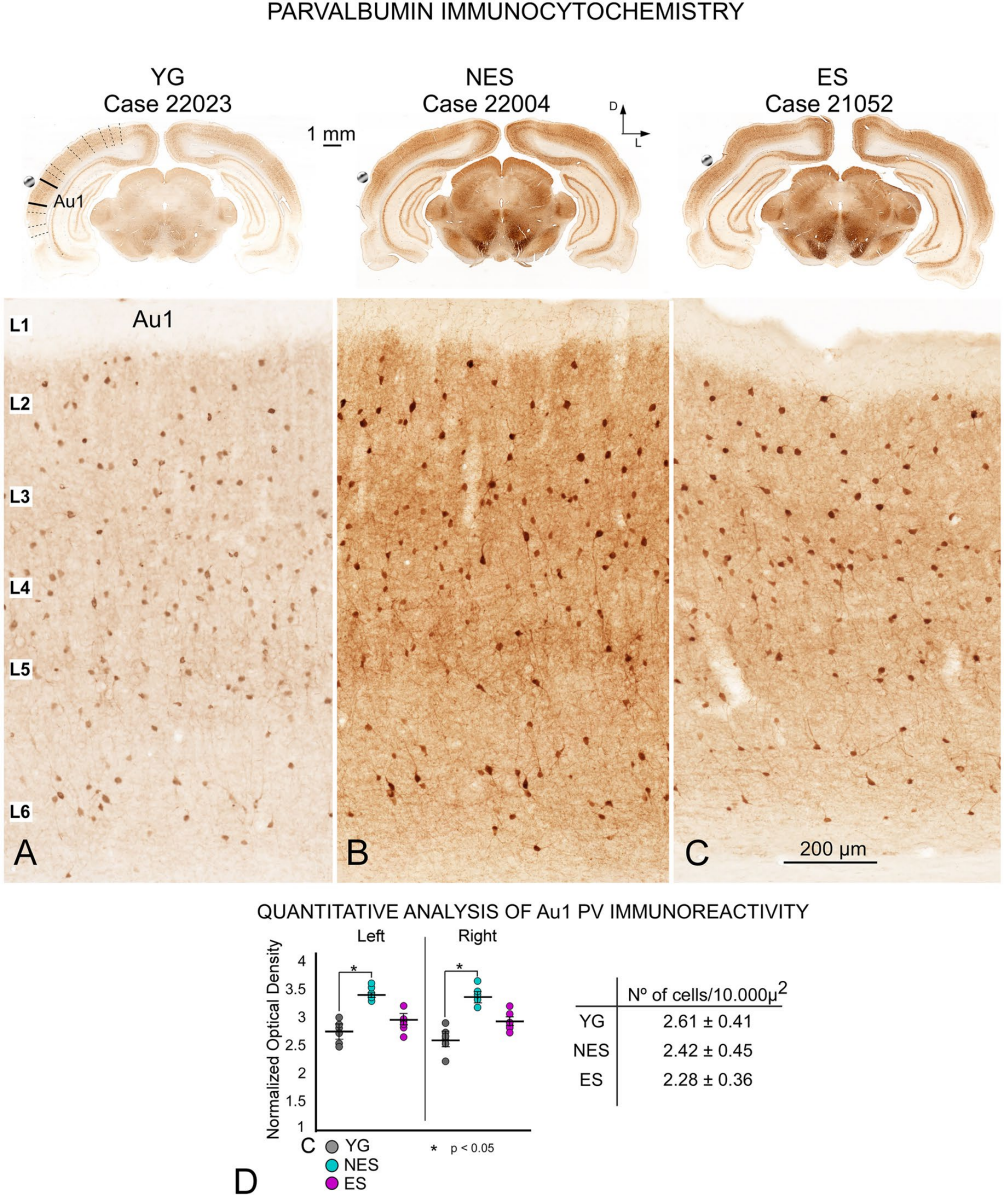
Parvalbumin immunocytochemistry. **A** YG. Panoramic view (top) shows differences in staining, defining the cytoarchitectural boundaries of the AC. At a higher magnification (bottom), positive neurons are distributed along all cortical layers, except layer 1, randomly distributed in supra-granular layers but more concentrated in the main axis of layers 5 and 6. Dendrites and terminal fields are more evident along layer 5. **B** NES. Density increases in all brain regions, as shown in the panoramic view at the top of the figure. At a higher magnification, the cytoarchitecture is well preserved, with increased staining in cell bodies, dendrites and terminal fields. **C** ES. Despite the lack of changes in cytoarchitectural or cell number changes, the density of reaction product slightly increases in neurons and neuropil along the AC. **D** OD values of Au1 in toto show that density is significantly higher in the NES group than in the YG and ES groups (left), with no significant differences in cell counts (right) between groups

Upon parvalbumin immunocytochemistry, fully labeled interneurons (cell bodies, dendrites and terminal fields) were detected across all layers of the AC, which enabled us to recognize almost all canonical neuronal types (Fig. 7). Because of their higher neuropil density, layers 3, 5 and 6 were better defined than layers 2 and 4 (Fig. 7A). To appropriately analyze global changes in staining, simultaneously immunocytochemically processed paired sections were imaged with consistent camera and microscopic illumination settings. Their images showed higher PV density in NES rats than in control and ES animals (compare A with C in Fig. 7B). OD values of the whole AC confirmed this observation, showing significantly higher global PV immunoreactivity in NES animals compared with YG in the right (p = 0.041) and left cortices (p = 0.038) (Fig. 7D left), with no significant differences in PV-positive neuron counts between groups (Fig. 7D right).

### GAD 67 immunocytochemistry

**Fig. 8:**
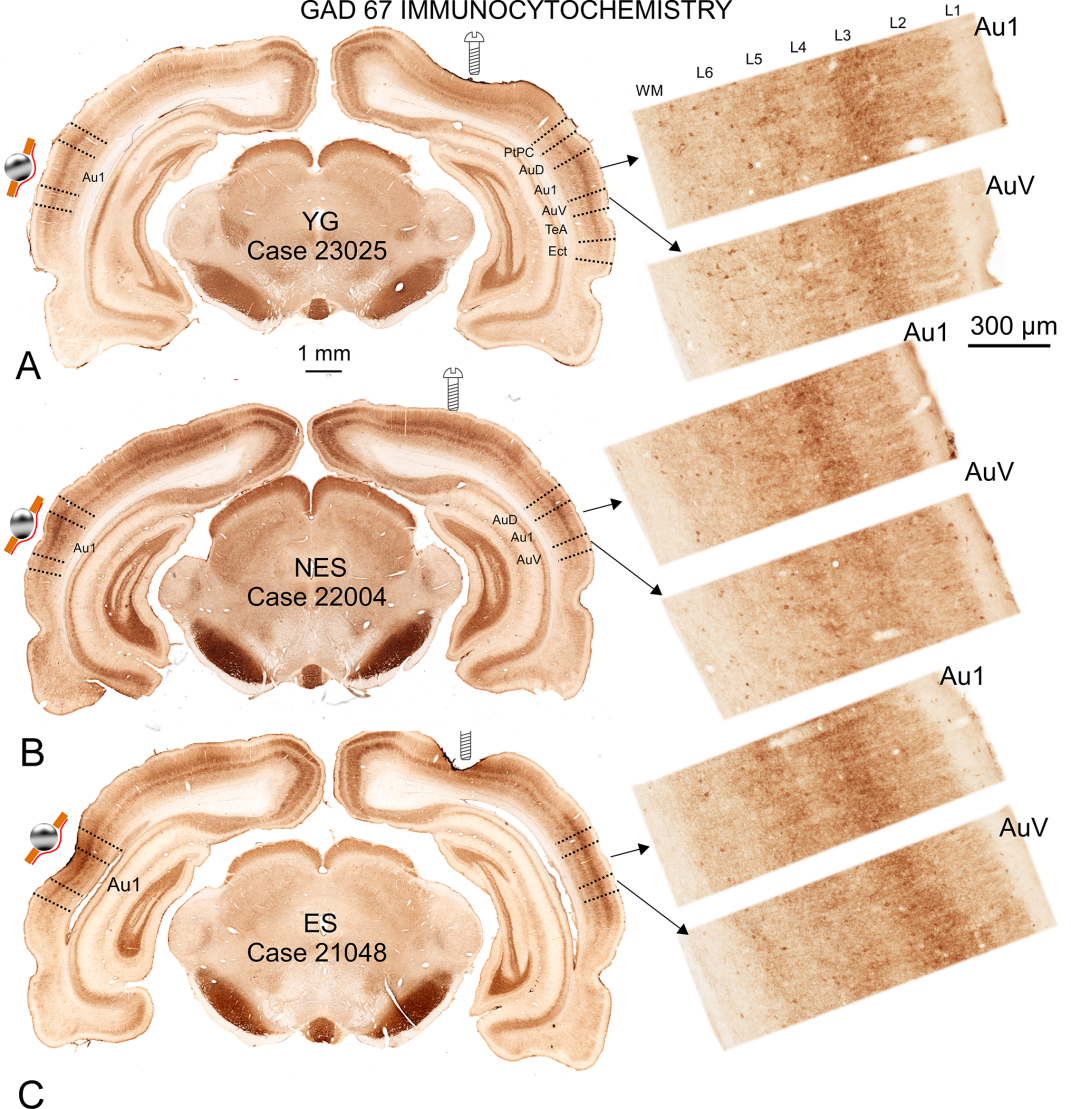
GAD 67 immunocytochemistry. The sphere indicates the localization of the epidural stimulation electrode in the section. The screw pinpoints the VC recording electrode. **A** YG. In a panoramic view (left of the panel), immunostaining shows a well-defined dense band crossing all auditory cortical regions, including AuD, A1, AuV, and TeA. The dorsal limit of the band matches the border between PtPC and AuD and the ventral border between the TeA and Ect (the limits on the right side of the panoramic view have been placed after superimposition of Paxinos and Watson map at IA 3.36). At a higher magnification (right side of the panel) immunoreactive cell bodies, dendrites and axonal terminal fields are found in all layers. The densest band corresponds to layer 3. **B** NES. Immunostaining density increases throughout the brain. However, the dense band in layer 3 is restricted to Au1. On the right side of the image, details at a higher magnification illustrate the missing labeling in AuV, in addition to fewer and less dense cell bodies and dendrites. **C** ES. A slight reinforcement of the meninge and increased density of neuropil is shown around stimulation area (silver ball symbol). The distribution, density and extension of labeling along the cortex are similar when comparing in layer 3 with A (YG). At a higher magnification, labeling in layer 3 of the secondary cortex AuV mirrors labeling in Au1. PtPC: parietal cortex, posterior area, caudal part; Aud: auditive dorsal; A1: primary auditory cortex; AuV: auditive ventral; TeA: temporal associative cortex; Ect: ectorhinal cortex

GAD 67 immunocytochemistry showed a better-defined band in layer 3 because its neurons and neuropil were strongly stained (Fig. 8). By analyzing the topographical distribution of this layer, we easily distinguished cortical cytoarchitectural subdivisions of the AC matching the landmarks of A1, AuD, AuV and TeA defined by Paxinos and Watson (Paxinos and Watson 2005) (Fig. 8 left dotted lines). In NES animals, immunoreactivity decreased in all layers of the secondary auditory cortices, especially in layer 3 (Fig. 8B details). These differences in staining were not detected in YG or ES animals (compare A with C in Fig. 8B). In ES rats, GAD 67 density increased approximately 1 mm in length and 0.5 to 0.7 mm in depth in the area of stimulation (Fig. 8C left side). On the contralateral side, in the secondary cortices, these animals showed a similar pattern to the immunostaining of YG animals (Fig. 8C).

### CAEPs

**Fig. 9:**
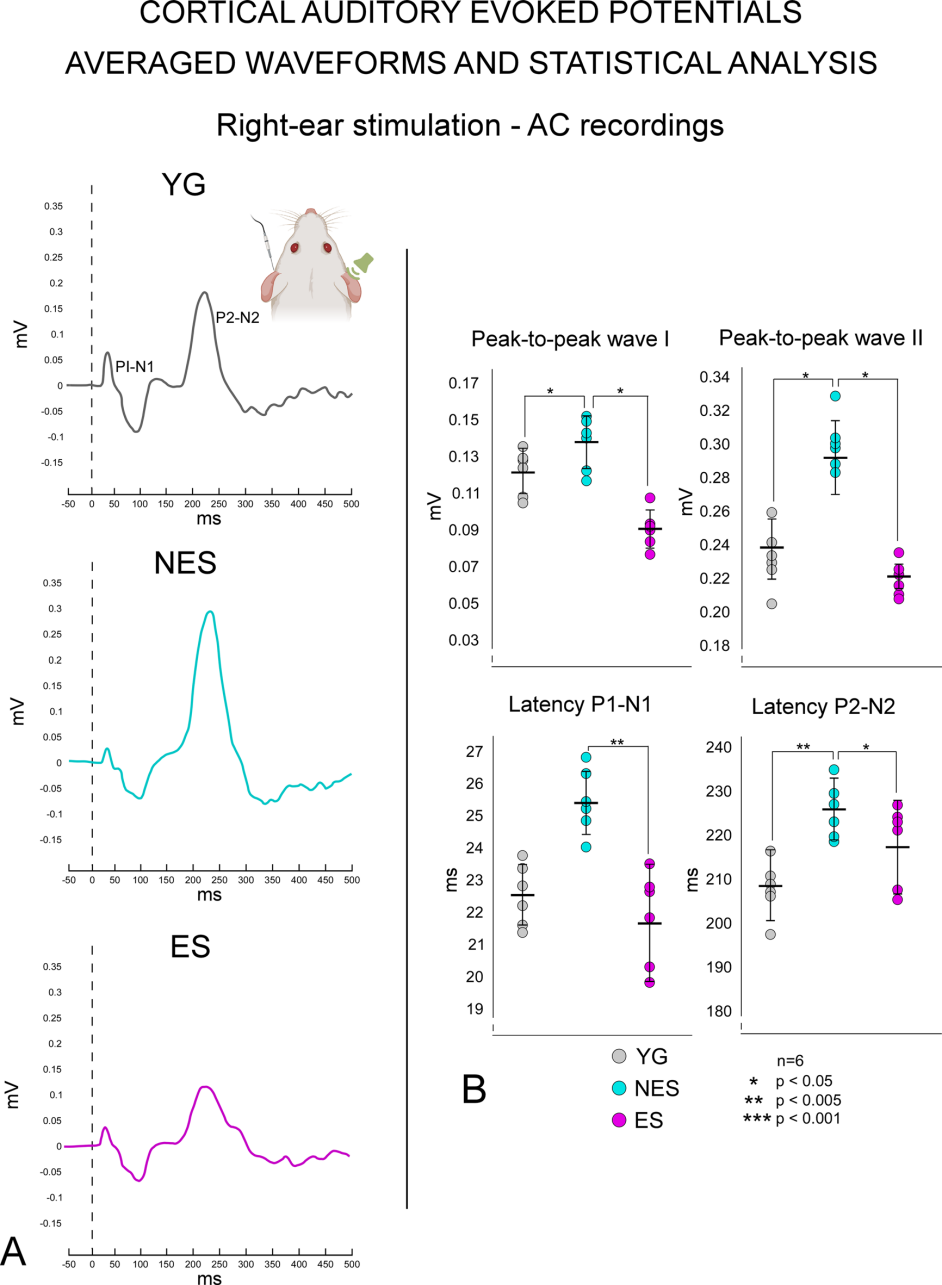
Results of averaged waveforms and statistical analysis of CAEPs after auditory stimulation (16 kHz – 50 dB). Recordings from the silver ball electrode placed at AC coordinates. Auditory stimulation in the right ear and recordings in the left AC (inset). **A** YG. Black line (top) shows a well-defined P1-N1 and P2-N2. Aged NES rats, (middle graph, cyan line) show a slight decrease of P1-N1 and a strong rebound of P2-N2. ES rats (bottom graph, magenta line) show a lower amplitude of P2-N2 than NES rats. **B** Statistical analysis of recordings shows significant differences in P1-N1 and P2-N2 amplitudes when comparing YG and ES with NES rats, with no significant differences between YG and ES rats. In the bottom, P1-N1 latencies are significantly longer in NES rats than in ES rats. P2-N2 latencies are also significantly longer in NES than in the other two experimental groups. Color codes: YG – gray, NES – cyan, ES – magenta

**Fig. 10:**
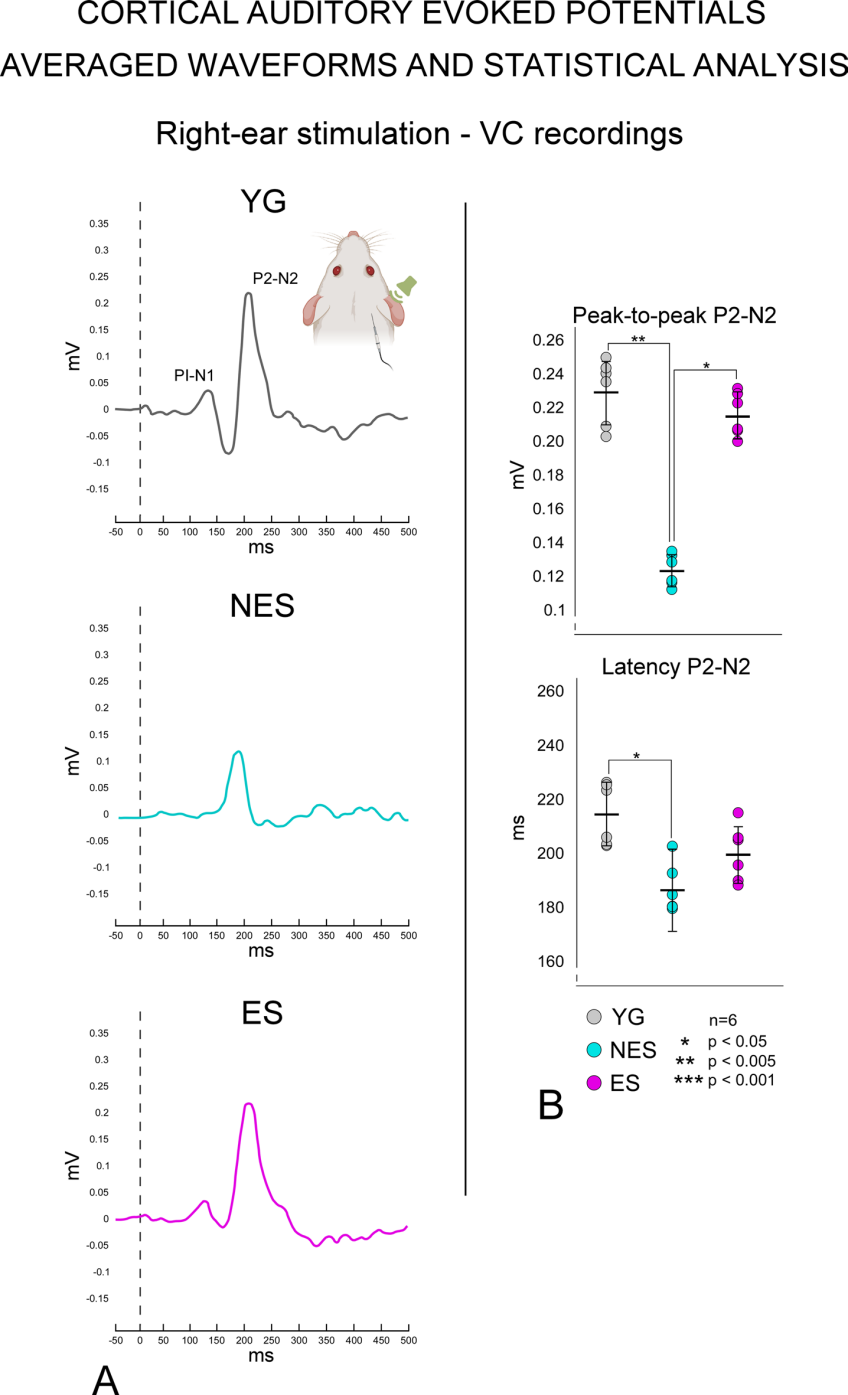
Averaged waveforms and statistical analysis of CAEPs after auditory stimulation (16 kHz – 50 dB) in the right ear and recordings in the right VC (contralateral side of electric stimulation) (inset). **A** Robust P1-N1 and P2-N2 (top) is shown in YG rats. NES rats do not show P1-N1 and have smaller P2-N2 (middle – cyan line). ES rats display a well-defined P1-N1 and a large P2-N2. **B** Because P1-N1 is not detected in NES in VC recordings, only P2-N2 can be analyzed. Significant differences are detected when comparing YG or ES with NES, but not when comparing YG with ES. Latencies significantly decrease only when comparing YG with NES. Color codes: YG – gray, NES – cyan, ES – magenta

We statistically compared waveforms of AC recordings from the three experimental conditions, namely YG (Fig. 9A top -black lines), NES (Fig. 8A middle—cyan lines), and ES (Fig. 9A bottom magenta lines), and the corresponding amplitudes and latencies values (Fig. 8B). In addition, VC recordings waveforms (Fig. 10A) and their numerical values of amplitudes and latencies were also statistically analyzed (Fig. 10B). After sound stimulation, averaged AC evoked potentials in YG animals showed waveforms with two well-defined waves at latencies of 22 ms (P1-N1) and 208 ms (P2-N2), with amplitudes of 0.121 and 0.237 mV, respectively (Fig. 9A black lines and B gray dots). Recordings of YG controls in the right VC electrode after sound stimulation showed 138 (P1-N1) and 214 (P2-N2) ms waves, with amplitudes of 0.048 and 0.230 mV, respectively (Fig. 10A, black lines; B, gray dots).

#### Aging effects

When comparing waveforms from the left side (AC recording electrode) between the YG and NES groups, we observed larger P2-N2 in the older animals (Fig. 9A compare top/black with middle/cyan lines). The peak-to-peak increase in P1-N1 (p > 0.04) and P2-N2 (p > 0.013) was significantly higher in NES than in YG (Fig. 9B). Although data suggest a delay in latencies of P1-N1 when comparing NES with young animals, no significant differences were identified, but the differences in P2-N2 were significant (p > 0.002) (Fig. 9B). In NES animals, evoked potentials recorded on the right side (VC electrodes) did not show any P1-N1 (Fig. 10A), but their P2-N2 amplitudes were significantly (p > 0.004) lower than those of YG animals, with a delay in latencies (p > 0.023) (Fig. 10B).

#### EE effects

In the YG and ES groups, the waveforms of both AC and VC recordings after electric stimulation showed P1-N1 and P2-N2 with similar shape and size (compare Fig. 9A and Fig. 10A top and bottom graphs). But in the NES group, P1-N1 (p > 0.032) and P2-N2 (p > 0.019) amplitudes of AC recordings were significantly lower than in the ES group (Fig. 9B), with a delay in latencies (p > 0.04 for P1-N1 and p > 0.04 for P2-N2). In ES animals, peak-to-peak values of VC recordings showed significantly higher amplitudes than those of NES animals (p > 0.043), with no significant differences from YG control animals (Fig. 10B). No significant differences in latencies of VC recordings were detected either when comparing ES and NES (Fig. 10B).

### Iba 1

**Fig. 11:**
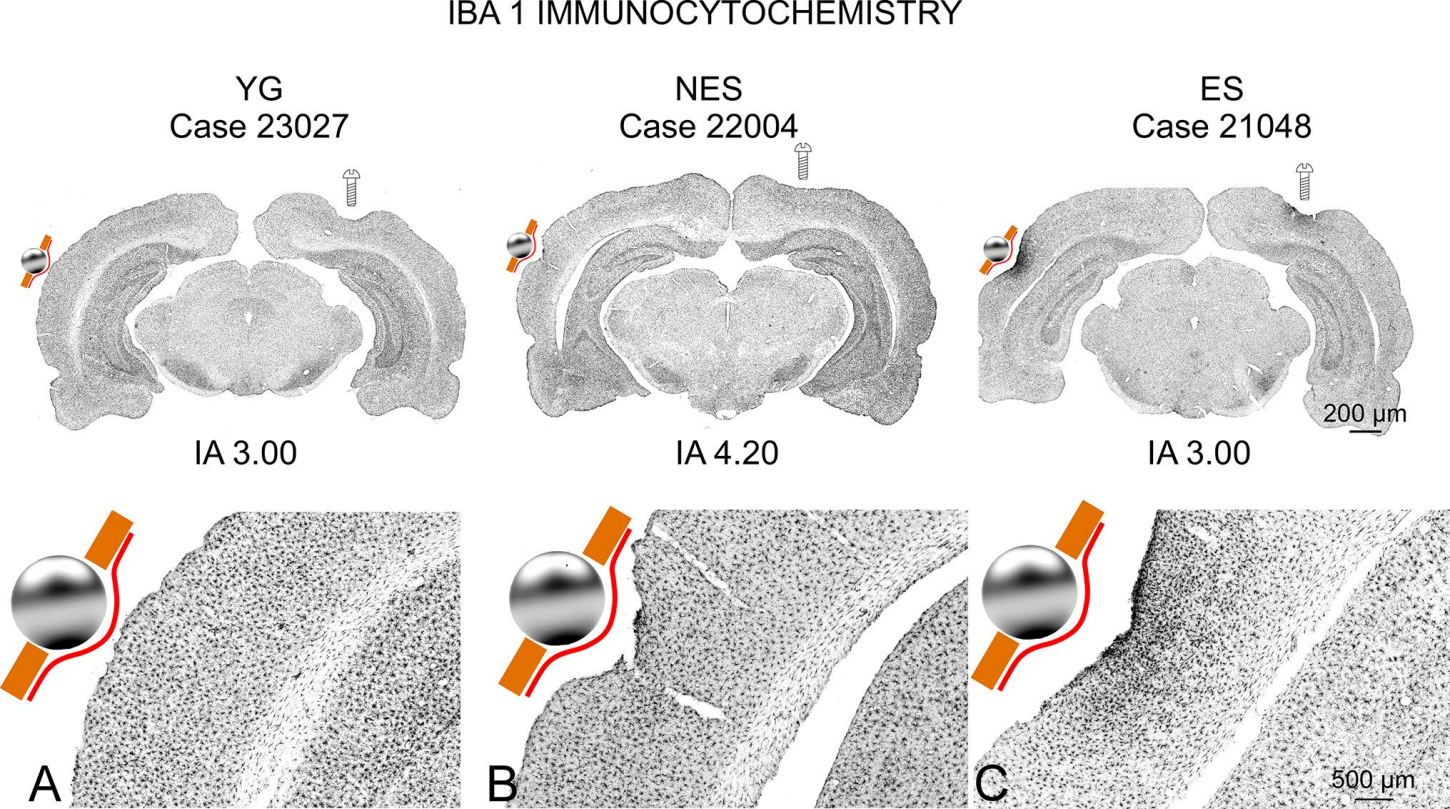
IBA 1 Immunocytochemistry. **A** YG. No potential inflammatory changes are detected by microglial labeling in sham-operated rats. **B** NES. No reactive microglia is detected in the electrode implantation area or along other nuclei in the section. **C** ES. A restricted glial reaction is observed along a surface band in the cortex. Note the increased density and packing of neurons overlapping with the electrode area implantation (sphere). The sphere symbol indicates the electrode position, as shown by a small depression in the section silhouette

The location of the electrode was easily recognized in sections by a small depression of the surface of the cortex, which enabled us to correctly position the electrode at the expected coordinates (Fig. 11). A small, restricted microglial reaction was observed in ES animals within a narrow surface band of approximately 500 to 700 µm, related to the electrode placement site (Fig. 11C). No evidence of reactive microglial cells was found outside the stimulation area.

## Discussion

In this study, we examined changes in cellular activity at the onset of ARHL in 17.13-month-old Wistar rats (NES) by immunocytochemistry. The results highlighted functional impairment (c-Fos; Figs. 2 and 3) and reduced synaptic plasticity (Arc and Glu 2/3; Figs. 4,5 and 6 respectively) in the AC. Inhibitory neurons were analyzed through PV immunocytochemistry, which showed no significant changes in the number of neurons between experimental groups but revealed global increased density in the AC of NES 18.13 old rats (Fig. 7). GAD 67 labeling decreased in both primary and secondary ACs, especially in the latter (Fig. 8). In the AC, recorded CAEPs showed significant increases in wave amplitudes and latencies (Fig. 9B). These data suggest that reduced cell activation and plasticity in the AC may increase excitability and delay auditory responses, underlying ARHL.

Applied at the onset of ARHL (defined at 16 months by monthly ABR recordings in Fernandez del Campo et al. 2024), multisession epidural DC anodal stimulation mitigated the loss of c-fos and Arc-labeled neurons (Figs. 2,3 and 4,5 respectively) and stabilized CAEP wave amplitudes and latencies. Additionally, no significant differences in PV (Fig. 7) and GAD67 (Fig. 8) immunostaining density or distribution were found between the ES and YG groups, indicating preservation of the AC inhibitory microcircuitry. These findings underscore the potential of electrical stimulation to delay the main features of AC impairment associated with aging.

Aging effects on 18.13-month-old rats.

In untreated aged animals (NES), the significant decrease in the normalized number of positive cells and mean area suggests reduced AC neuronal activation (Fig. 3). This decrease should be related to threshold elevations of approximately 15 dB recorded at the same time point of the protocol (Supp Mat.). If so, the decline in altered lemniscal stimulation following ARHL may explain the loss of AC activation. However, in our animal model, metabolic (Wang et al. 2016) and inflammatory (Pawate et al. 2004; Di Stadio and Angelini 2019) changes observed in the aging brain may act in combination with altered ascending inputs. Previous findings of our researched group demonstrated a correlation between cochlear input loss (ensured by a rat bilateral cochlear ablation model) and reduced c-fos and Arc immunoreactivity in the AC (Pernia et al. 2020). In this model, the decrease of immunoreactive cells in the AC matched that observed in our aged rats (see Pernia et al. 2020, its Figs. 2 and 4). In terms of auditory pathway activation, these data indicate that early genes strongly react to changes in lemniscal AC activation. Accordingly, the loss of c-fos observed in our aged rats aligns with threshold elevations (ARHL) shown by ABR recordings around 15–20 dB (SI).

Arc downregulation impairs homeostatic scaling of AMPARs after chronic neuronal activity blockade with TTX in high-density cortical cultures (Shepherd and Bear 2011). In addition, Arc is involved in activity-dependent dendritic plasticity (Guzowski et al. 2000), regulating homeostatic synaptic plasticity (McCurry et al. 2010) and dendritic spine morphology by controlling cortical neuron excitability (Peebles et al. 2010). In our NES rats, Arc immunoreactivity loss coincided with reduced AMPA Glu 2/3 staining of hypolemmal density in the soma and dendrites of pyramidal neurons (see arrows in Fig. 5). Based on these results, aging may impair synaptic plasticity and decrease homeostatic regulation of AMPA glutamate receptors in the AC.

Loss of inhibition is one of the most important brain alterations in aging, with broad scientific consensus (Backoff and Caspary 1994; Ling et al. 2005; Burianova et al. 2009; Martin del Campo et al. 2012; Gao et al. 2015; Ouda et al. 2015; Rozycka and Liguz-Lecznar 2017; Ueno et al. 2019; Rogalla and Jannis Hildebrandt 2020; Wirak et al. 2022). In our PV immunocytochemistry analysis, we found no significant cell count differences, so at least at early aging (18.13 months), this interneuron population is not severely affected. The PV reaction product did increase across the cortex in aged animals (Fig. 7), as in the AC of 22-month-old Long–Evans rats (Ouda et al. 2008) despite differences in the strains (Long Evans vs Wistar) and age of the animals (18.13 vs 22 months). But unlike those authors, we did not detect areas devoid of positive neurons. Overall, our results did not reveal major differences in PV-positive cell counts. Most PV immunoreactive neurons, particularly fast-spiking and basket cells, co-localize with GABA, implying that changes in immunoreactivity of this calcium-binding protein potentially compromise the microcircuits in charge of the fine regulation of pyramidal cell responses (Celio et al. 1986; Kosaka et al. 1987; DeFelipe and Jones 1992; Kawaguchi and Kubota 1998; Ouda et al. 2008). Because calcium-binding proteins are upregulated in aging cells with elevated calcium concentrations (Raynard et al. 2022), the increase in PV immunoreactivity may be related to calcium influx, which is known to trigger cell death signaling in aging (Martin et al. 2023). Accordingly, this increase in PV staining with aging may be an early sign of cell death although specific counts must be performed in older rats to support this hypothesis.

The decrease in GAD labelling of secondary cortices (AuV and AuD) of the AC with aging observed in this study (Fig. 8B) corroborates previous findings in Long-Evans and Fischer 344 rats by western blot analysis (Burianova et al. 2009) and in Sprague Dawley rats by immunocytochemistry and in situ hybridization (Ling et al. 2005). In response to aging, the oligomeric reconfiguration of GABA receptors (especially GABA A) in the primary AC suggests a net reduction of functional inhibition in neuronal responses (Caspary et al. 2008). In PV interneurons, selectively removing one allele of the GAD67 gene, Gad1, induces substantial deficits in transmission in prefrontal cortex and increases pyramidal cell excitability (Lazarus et al. 2015). Notwithstanding these differences in strains and methods, the decrease in GAD 67 labelling assessed in our NES rats (Fig. 7) may thus align with the increase in excitability detected in CAEPs (Fig. 9).

AC hyperexcitability has been associated with decreased GABA inhibition in various hearing impairment models (Kotak 2005; Salvi et al. 2017; Herrmann and Butler 2021; Gürkan et al. 2024). In NES rats, the loss of GAD 67 immunoreactivity differed between the A1 and secondary cortices, with a decrease of labeling more evident in layer 3 (Fig. 8). During aging, cross-modal compensation implies a deep functional and anatomical reorganization (Lomber et al. 2010; Wong et al. 2014; Cardon and Sharma 2018), as shown by reduced intermodal connectivity in Mongolian gerbils (Henschke et al. 2018). So, from a connectivity standpoint, the decrease in GAD immunoreactivity in secondary cortices (AuD, AuV and TeA) may reflect either a passive effect after loss of inputs (residual plasticity) or a deep plastic reorganization of inhibitory intermodal connections, which could also help to explain increases in CAEP responses.

In our physiological analysis, CAEPs recorded in the VC of YG rats displayed well-defined P1-N1 and robust P2-N2, in contrast to the loss of P1-N1 and small-amplitude of P2-N2 observed in NES rats. This reduced P2-N2 amplitude, without P1-N1, suggests a decrease in sound responsiveness in the VC of NES rats and likely reflects an intermodal disbalance of VC and AC feedback regulation. This potential change in cross-modal regulation with aging may promote the decrease in GAD inhibition may also contribute to an altered AC response to sound.

Based on these findings, our working hypothesis is that ARHL (shown by ABRs) and loss of (i) neuronal activation (c-Fos), (ii) synaptic plasticity regulation (homeostatic compensations—ARC and Glu 2/3) and (iii) inhibition (GAD 67) in the AC of our NES rats, together with the increased CAEP response to sound, reflecting an unbalanced regulation of excitation and inhibition. Such an imbalance is most likely induced by the decreased stimulation of cochlear inputs, by metabolic changes and by altered cross-modal regulation.

### Effects of electric stimulation

In our ES group, we observed a local increase in the number and size of c-fos immunoreactive neurons under the area of the stimulation electrode and a significant increase in optical density of its reaction product. These results demonstrate that our protocol effectively induces a bilateral neuronal network activation. Furthermore, increases in Glu 2/3 AMPA receptors and Arc immunocytochemistry suggest that our stimulation protocol can also induce sustained activation of the AC. Sustained activation is supported by changes in auditory thresholds 2 days after multisession stimulation (Colmenarez-Raga et al. 2019) and by all functional changes in animals euthanized 5 days after the last stimulus (see M&M). At the onset of auditory aging, this sustained activation, for two weeks, may explain anatomical and functional preservation of the AC in ES rats.

The efferent system has an otoprotective effect during aging (Boero et al. 2020). AC activation through a similar protocol of electric stimulation also indirectly helps to preserve auditory responses by threshold stabilization upon descending pathway regulation (Fernández del Campo et al. 2024), as confirmed in this study (SI). As shown by histological analysis of these experiments, the cochlea is better preserved and ARHL inflammation is decreased in stimulated animals at 18 months of age (Fuentes-Santamaría et al. 2024). Based on these results, cortical aging compensation in ES rats should be related to preservation of the cochlea and cochlear inputs and, consequently, to improved regulation of the ascending pathway to the AC.

Anodal DC stimulation may also directly enhance cortical responses by increasing neuronal network activation (Nitsche and Paulus 2000; Nitsche et al. 2002; Lang et al. 2004), mitigating trophic, metabolic or inflammatory changes associated with aging (Mattson, Mark P. and Arumugam 2018). Because most of these potential effects depend on the activity of cortical neuronal networks, the indirect effect of auditory input preservation induced by our protocol may also help to stabilize the AC, as previously observed in ES rats (Fernandez del Campo et al. 2024). Enhancing the CNS field activation may induce trophic factor activation (Fritsch et al. 2010; Kim et al. 2017). For example, TrkB receptor inhibition in the hippocampus decreases the positive effects of DC stimulation, so neuronal function and survival after electric stimulation may also be promoted by trophic factors, such as brain-derived neurotrophic factor (BDNF; Podda et al. 2016). Supporting this assumption, the mRNA levels of both c-fos and BDNF increase upon repetitive stimulation of the sensorimotor cortex of rats (Kim et al. 2017). In present study, the complexity of our protocol prevented us from analyzing trophic factors that synergistically upregulate c-fos and BDNF. But this hypothesis is also supported by the parallel transcription regulation of Ca^2+^/cAMP-response element binding protein (CREB) and serum response factor (SRF; Robertson et al. 1995; Bito et al. 1996; Flavell and Greenberg 2008). Dependent on calcium inflow, calcium sensors and calcium buffering, such a pathway may enable a mechanism of compensation for decreased synaptic plasticity and homeostatic regulation (Denaxa et al. 2018; Hadler et al. 2024).

A limitation of this study is the lack of consensus on the increase in excitability with aging observed in human CAEP analysis, most likely due to differences in stimulus and recording parameters, electrode placement, audibility, and age-related characteristics (Polen 1984; Wall et al. 1991; Bertoli et al. 2011; Campbell and Sharma 2013; McClannahan et al. 2019). Nevertheless, previous studies in rodents have shown that increased excitability is a common effect of aging and hearing loss (Kotak 2005; Salvi et al. 2017; Herrmann and Butler 2021; Gürkan et al. 2024). And despite differences in the stimulus and location of the electrodes, our CAEPs recorded in YG rats display wave forms and amplitudes compatible with previous results from other research groups (Simpson and Knight 1993; Takahashi et al. 2004, 2007). Case in point, our CAEP recordings in the AC highlighted a significant increase in excitability in NES rats, in contrast to stabilized amplitudes and latencies in ES rats, with only nonsignificant differences from YG rats.

Another limitation of our analysis is related to the immunocytochemical quantification of GAD67-immunostained sections. The labeling pattern included dense, immunopositive terminal fields that overlapped with positive neurons, obscuring their soma and preventing accurate density threshold segmentation. Similarly, Glu2/3-immunoreactive sections displayed tightly packed pyramidal neurons, most of which were superimposed along the thickness of the section. The high density of neurons in layer 6 further prevented accurate measurements. For these reasons, the immunoreactivity of these two antibodies was described based solely on direct microscopic observation.

Taken together, these results support our hypothesis that multisession electric stimulation at the onset of aging delays the imbalance between excitation and inhibition and increases in AC responses to sound. Concurrently, the global increase in PV immunoreactivity is slightly lower in ES than in NES, suggesting a potential decrease in calcium inflow and, thus, signaling interactions with almost all study markers (Martin et al. 2023). However, our study focused on activity-dependent changes of the cortex. Therefore, further research should be conducted to analyze the calcium signaling pathway in order to better assess the effect of DC stimulation on trophic factors and on the activation of cell-death signaling cascades.

## Conclusion

During early sensory decline, multisession DC stimulation of the auditory cortex delays central ARHL by minimizing the loss of inhibition and by preventing increases in cortical excitability in Wistar rats.

## Supplementary Information

Below is the link to the electronic supplementary material.

Supplementary file1 Statistical analysis of ABR thresholds of YG (gray), NES (cyan) and ES (magenta) groups. Although YG shows significantly lower thresholds than the other two experimental groups, these thresholds are more stabilized in ES than in NES.(TIF 14942 KB)

## Data Availability

No datasets were generated or analysed during the current study.

## Abbreviations

ABR: Auditory brainstem response
AC: Auditory cortex
AMPA: α-Amino-3-hydroxy-5-methyl-4-isoxazolepropionic acid
ARHL: Age-related hearing loss
AuD: Dorsal auditory cortex
AuV: Ventral auditory cortex
CAEP: Cortical auditory evoked potential
DC: Direct current
Ect: Ectorhinal cortex
ES: Electrically stimulated
GABA: Gamma aminobutyric acid
GAD: Glutamate decarboxylase
NES: Non-electrically stimulated
OD: Optical density
PtPC: Posterior parietal cortex
ROS: Reactive oxygen species
TeA: Temporal associative cortex
VC: Visual cortex
P1-N1: Wave one
P2-N2: Wave two
Y: Young
