# Supplementary figures and images for: Anodal direct current stimulation of the auditory cortex at the onset of presbycusis delays cortical aging
Source: Brain Struct Funct. 2025 Apr 25;230(4):56. doi: 10.1007/s00429-025-02912-w (PMC12031871; doi:10.1007/s00429-025-02912-w)

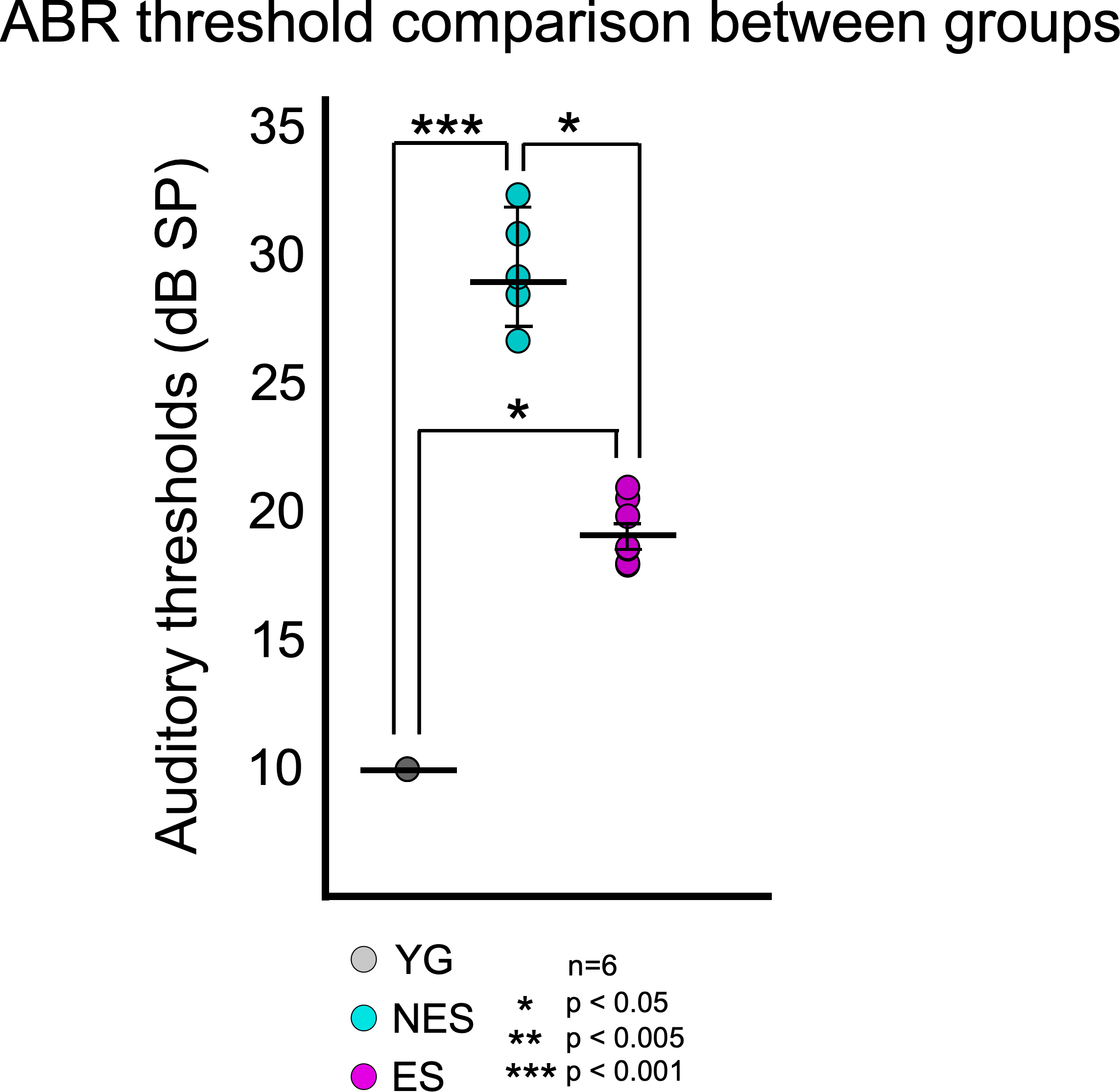

Supplement: Supplementary file 1 — Supplementary file1 Statistical analysis of ABR thresholds of YG (gray), NES (cyan) and ES (magenta) groups. Although YG shows significantly lower thresholds than the other two experimental groups, these thresholds are more stabilized in ES than in NES.(TIF 14942 KB) [file 429_2025_2912_MOESM1_ESM.tif]
